# Supplementary material for: Dendrochronological evidence for long-distance timber trading in the Roman Empire
Source: PLoS One. 2019 Dec 4;14(12):e0224077. doi: 10.1371/journal.pone.0224077 (PMC6892532; doi:10.1371/journal.pone.0224077)
Supplement: S1 Table — Species: Quercus sp. Tucson format. (PDF) [file pone.0224077.s001.pdf]

Series code: 0000C1

0000C1	-0243	63	68	62							
0000C1	-0240	81	65	50	47	34	36	37	40	62	72
0000C1	-0230	72	66	70	66	86	110	96	87	98	98
0000C1	-0220	86	90	72	74	86	101	87	106	103	84
0000C1	-0210	89	101	78	103	112	89	95	94	88	70
0000C1	-0200	74	70	78	80	67	103	70	54	61	74
0000C1	-0190	89	98	70	83	65	70	81	92	85	88
0000C1	-0180	64	87	80	51	48	42	58	91	78	68
0000C1	-0170	87	102	108	106	114	96	70	78	68	80
0000C1	-0160	70	68	60	58	72	58	62	58	80	72
0000C1	-0150	59	86	74	74	82	104	99	81	70	68
0000C1	-0140	63	115	86	94	86	98	96	102	94	115
0000C1	-0130	82	99	122	152	105	116	99	74	72	69
0000C1	-0120	86	90	85	103	81	82	86	67	90	74
0000C1	-0110	73	98	88	70	85	78	78	86	88	73
0000C1	-0100	76	88	95	90	84	100	84	57	70	59
0000C1	-0090	67	56	72	88	66	62	53	44	36	52
0000C1	-0080	56	67	80	82	68	78	81	77	65	58
0000C1	-0070	75	67	77	72	92	69	76	82	79	78
0000C1	-0060	88	78	85	74	65	86	74	55	70	90
0000C1	-0050	86	111	78	66	78	58	82	80	82	92
0000C1	-0040	61	87	88	66	68	74	77	75	93	92
0000C1	-0030	84	84	86	98	88	106	72	97	96	146
0000C1	-0020	106	150	70	76	98	95	102	103	92	92
0000C1	-0010	100	136	104	101	102	100	98	106	149	128
0000C1	0000	100	112	83	86	116	99	90	88	94	96
0000C1	0010	110	100	94	87	118	91	142	99	108	102
0000C1	0020	110	76	81	76	96	121	136	144	111	115
0000C1	0030	108	95	104	111	123	120	90	73	96	126
0000C1	0040	124	999								

Series code: 000C17

000C17	-0133	145	118	101							
000C17	-0130	80	96	106	144	134	122	123	88	98	91
000C17	-0120	94	86	84	80	84	82	100	86	86	88
000C17	-0110	133	105	105	100	122	114	115	114	116	130
000C17	-0100	105	93	139	134	90	116	116	112	108	84
000C17	-0090	96	110	103	139	86	87	72	0	58	98
000C17	-0080	78	119	110	138	140	162	161	122	67	71
000C17	-0070	75	78	73	72	85	70	72	80	81	72
000C17	-0060	86	86	100	110	89	104	95	76	96	96
000C17	-0050	94	138	76	68	94	78	126	98	116	117
000C17	-0040	68	85	74	71	88	82	74	62	89	90
000C17	-0030	107	101	112	105	108	76	83	92	78	114
000C17	-0020	59	83	71	72	66	70	96	74	90	86
000C17	-0010	80	80	84	98	101	82	106	98	96	104
000C17	0000	111	86	98	106	105	98	77	74	80	112
000C17	0010	108	94	87	83	124	97	156	108	117	123
000C17	0020	91	87	90	90	99	82	116	106	100	148
000C17	0030	108	90	112	140	186	130	98	78	81	64
000C17	0040	74	999								

Series code: 000C22

000C22	-0076	57	73	76	60	67	85				
000C22	-0070	72	69	62	64	83	85	76	82	72	58
000C22	-0060	60	66	95	106	103	111	82	82	81	123
000C22	-0050	140	212	196	148	183	180	198	144	156	197
000C22	-0040	130	144	116	162	126	146	138	103	136	140

000C22	-0030	138	141	162	111	147	114	142	134	132	222
000C22	-0020	147	169	108	174	129	134	111	146	161	128
000C22	-0010	134	161	135	160	184	181	203	218	228	298
000C22	0000	224	179	157	148	156	186	160	151	118	132
000C22	0010	136	108	96	96	116	108	146	118	132	156
000C22	0020	132	127	120	84	999					

Series code: 000C27

000C27	-0184	155	125	141	129						
000C27	-0180	108	144	120	96	61	89	102	106	81	78
000C27	-0170	107	93	114	97	109	105	81	120	99	97
000C27	-0160	113	100	79	97	114	112	107	88	95	139
000C27	-0150	99	119	129	128	122	163	151	100	107	83
000C27	-0140	71	129	92	99	117	94	129	122	97	109
000C27	-0130	68	79	108	117	108	85	57	69	84	78
000C27	-0120	76	75	75	83	84	91	90	90	71	46
000C27	-0110	66	66	78	80	105	93	92	91	83	72
000C27	-0100	79	70	74	64	74	78	54	54	54	50
000C27	-0090	56	60	62	66	54	48	39	35	999	

Series code: 000C29

000C29	-0092	214	178								
000C29	-0090	186	204	178	154	147	141	144	90	58	99
000C29	-0080	106	118	122	124	142	119	136	111	146	142
000C29	-0070	148	157	157	122	144	98	86	104	101	94
000C29	-0060	101	92	82	81	86	92	88	82	88	138
000C29	-0050	112	140	129	118	116	110	144	107	112	112
000C29	-0040	86	97	95	104	112	88	97	94	99	118
000C29	-0030	118	114	125	106	88	101	114	119	110	145
000C29	-0020	108	128	71	96	66	74	82	76	98	88
000C29	-0010	106	94	96	112	119	122	122	132	108	88
000C29	0000	99	115	88	59	56	74	76	80	68	66
000C29	0010	69	60	78	78	72	82	84	111	112	130
000C29	0020	114	116	999							

Series code: 000C23

0000C3	-0279	141	176	138	161	178	210	219	254	165	
0000C3	-0270	164	175	140	146	151	137	148	115	129	146
0000C3	-0260	141	120	138	148	116	104	118	108	116	108
0000C3	-0250	116	147	112	120	102	102	95	102	124	108
0000C3	-0240	108	96	85	105	130	106	117	110	108	102
0000C3	-0230	110	106	100	106	116	122	105	109	118	114
0000C3	-0220	94	132	90	80	107	110	90	106	103	78
0000C3	-0210	107	110	127	124	100	104	114	121	94	94
0000C3	-0200	91	90	81	77	70	86	77	82	65	74
0000C3	-0190	100	109	99	105	102	103	85	87	93	92
0000C3	-0180	68	108	86	76	61	80	85	103	103	79
0000C3	-0170	88	103	95	92	95	117	70	95	98	77
0000C3	-0160	83	80	78	77	65	77	72	62	71	70
0000C3	-0150	63	70	72	62	69	77	74	69	74	68
0000C3	-0140	61	80	62	72	66	60	73	72	66	86
0000C3	-0130	79	68	78	76	78	86	72	68	76	67
0000C3	-0120	86	79	69	72	74	80	81	70	88	70
0000C3	-0110	86	90	68	84	81	88	74	79	67	68
0000C3	-0100	80	69	94	81	98	104	74	76	68	76
0000C3	-0090	58	62	70	79	77	76	74	50	54	74
0000C3	-0080	73	82	88	90	75	78	82	84	66	60
0000C3	-0070	62	72	68	58	72	72	62	68	80	72
0000C3	-0060	72	66	64	70	68	76	66	68	74	72
0000C3	-0050	70	90	84	72	72	68	92	68	82	82

```
0000C3 -0040    61    82    68    74    66    74    68    64    70    76
0000C3 -0030    67    66    74    64    74    62    68    82    75    94
0000C3 -0020    70    92    59    72    72    78    79    76    78    67
0000C3 -0010    78    72    81    78    86    64    84    91    88    75
0000C3  0000    80    76    72    76   999

Series code: 000C37
000C37 -0062   182   161
000C37 -0060   170   185   142   166   127   118   126    87   136   154
000C37 -0050   160   177   130   100    90    92   109    89    84   108
000C37 -0040    72    95    79    88    82    96    98    86   108    92
000C37 -0030    84    97   109   111   106   105   109   108    87   128
000C37 -0020    87   109    61    71    67    59    63    93    87    68
000C37 -0010    68    85    63    71    71    60    66    80   110   103
000C37  0000    92    85    85    80    80    87    89    96    83    81
000C37  0010    86    87   100    94   105    78   112   100   106   121
000C37  0020   111    98    85    72    94    81    77    72    73    96
000C37  0030   100    90    78    78    94   999

Series code: 000C54
000C54 -0048   226   146   161   208   188   150   114   176
000C54 -0040   110   132   115   147   136   141   124   110   116   105
000C54 -0030   119    99    90    80    77    80    84    92    91   123
000C54 -0020    94   110    68    49    50    48    43    52    64    68
000C54 -0010    59    64    64    69    99    92    86   109    98   100
000C54  0000   101    95    91    76    86    88    87    75    86    90
000C54  0010    88    89    74    94    75    86    91    88    87    78
000C54  0020    73    52    62    66    68    55    70    76    52    50
000C54  0030    56   999

Series code: 000C57
000C57 -0070   119   118   123    99   131   116   115   171   212   157
000C57 -0060   141   124   140   106    90   105    84   100   138   178
000C57 -0050   164   218   165   150   164   172   149   116   120   150
000C57 -0040   115   158   114   106   110   118   100    86    94   114
000C57 -0030   102   106   144   124   115    98   122    90    68   102
000C57 -0020    82    74    58    62    49    55   101    95    95    64
000C57 -0010    78   999

Series code: 000C59
000C59 -0061   284
000C59 -0060   299   240   305   284   214   225   191   142   219   162
000C59 -0050   193   319   325   272   270   254   260   192   310   252
000C59 -0040   163   206   142   166   186   184   160   125   140   176
000C59 -0030   208   205   200   144   139   104   110    87   108   180
000C59 -0020   110   152   108   134   131   123   107   139   166   122
000C59 -0010   148   116    92   144   149   125    99   133   999

Series code: 0000C7
0000C7 -0254   229   210   208   185
0000C7 -0250   193   171   184   149   135   113   103   154   142   138
0000C7 -0240   124   125   104   160   114   122   156   175   190   193
0000C7 -0230   148   132   132   122   185   132   172   166   148   130
0000C7 -0220   102   129   111   134   156   167   150   155   147   118
0000C7 -0210   125   156   153   125   121   112   104   119    96    99
0000C7 -0200    82   106   107   128    86    98    73    86    82    94
0000C7 -0190    98   124    88   117   112   100    98   102   109    91
0000C7 -0180    77   115    81    86    59    89    79   109   104    84
0000C7 -0170    97    87    84    84    86    91    80    99    88    84
0000C7 -0160    78    76    88    87    99    85    66    61    90    73
```

0000C7	-0150	56	72	72	64	62	90	78	80	81	78
0000C7	-0140	62	90	58	73	72	68	86	64	81	78
0000C7	-0130	71	77	77	82	65	61	57	58	59	60
0000C7	-0120	61	61	62	61	63	57	61	55	45	63
0000C7	-0110	69	59	62	61	65	66	64	63	61	63
0000C7	-0100	58	63	67	66	66	55	55	55	56	56
0000C7	-0090	52	52	58	55	51	54	40	39	44	48
0000C7	-0080	52	52	50	52	48	52	53	41	44	40
0000C7	-0070	48	44	35	33	43	48	40	42	39	47
0000C7	-0060	43	38	43	51	37	37	37	39	46	41
0000C7	-0050	44	46	43	41	47	45	51	46	41	45
0000C7	-0040	47	64	51	46	41	50	42	43	45	57
0000C7	-0030	51	37	43	45	43	49	50	46	42	56
0000C7	-0020	44	55	40	49	49	44	42	43	45	52
0000C7	-0010	41	55	58	53	48	46	51	55	59	55
0000C7	0000	60	54	53	55	53	56	42	60	52	54
0000C7	0010	48	49	62	43	71	999				

Series code: 000C24

000C24	-0112	110	81								
000C24	-0110	101	101	98	106	117	95	87	115	112	106
000C24	-0100	107	88	110	119	107	117	109	111	108	82
000C24	-0090	86	89	88	94	78	89	81	81	89	103
000C24	-0080	107	97	96	108	114	105	120	98	76	81
000C24	-0070	96	104	84	72	86	100	91	113	112	100
000C24	-0060	108	115	113	109	99	104	103	107	999	

Series code: 000C58

000C58	-0138	93	74	80	103	83	88	96	101		
000C58	-0130	93	79	101	123	139	129	88	108	95	88
000C58	-0120	149	93	92	92	111	116	116	98	103	98
000C58	-0110	99	74	88	84	95	92	82	98	96	71
000C58	-0100	110	87	103	160	149	123	169	153	120	74
000C58	-0090	108	101	93	84	89	70	89	74	65	93
000C58	-0080	110	97	72	93	94	85	132	122	127	84
000C58	-0070	101	109	88	85	125	106	65	57	65	60
000C58	-0060	71	66	99	91	77	87	78	74	73	87
000C58	-0050	112	163	138	104	143	79	95	81	90	155
000C58	-0040	101	110	111	91	121	121	135	121	110	108
000C58	-0030	94	108	131	125	99	90	107	95	94	125
000C58	-0020	77	77	70	89	105	93	95	103	91	82
000C58	-0010	95	84	84	78	81	107	102	114	112	106
000C58	0000	98	105	109	999						

Mean chronology

Mean s	-0279	141	176	138	161	178	210	219	254	165	
Mean s	-0270	164	175	140	146	151	137	148	115	129	146
Mean s	-0260	141	120	138	148	116	104	155	142	147	134
Mean s	-0250	142	155	136	130	113	106	98	97	105	96
Mean s	-0240	100	89	75	93	88	81	93	95	106	108
Mean s	-0230	102	95	94	93	118	119	115	112	116	111
Mean s	-0220	92	115	87	88	108	118	101	116	112	88
Mean s	-0210	103	116	113	116	109	100	104	110	92	85
Mean s	-0200	82	85	85	88	72	95	73	72	67	78
Mean s	-0190	95	108	88	100	91	92	96	96	100	101
Mean s	-0180	80	113	93	78	58	75	84	102	90	77
Mean s	-0170	95	97	101	95	101	102	76	100	90	85
Mean s	-0160	87	82	79	82	90	85	77	68	85	90
Mean s	-0150	70	87	90	85	86	111	103	83	84	74
Mean s	-0140	65	106	78	85	88	84	98	95	91	97

Mean s	-0130	76	83	98	113	99	93	78	73	78	74
Mean s	-0120	84	78	75	80	79	81	85	75	80	71
Mean s	-0110	87	84	83	83	96	88	84	93	89	85
Mean s	-0100	87	79	98	99	93	98	90	88	92	77
Mean s	-0090	82	91	90	95	80	81	76	65	65	85
Mean s	-0080	87	91	91	100	98	97	107	91	82	78
Mean s	-0070	88	93	86	76	96	86	77	88	96	96
Mean s	-0060	102	97	108	104	90	100	88	80	102	114
Mean s	-0050	119	167	150	121	135	125	139	109	123	142
Mean s	-0040	96	118	99	105	108	111	106	92	103	109
Mean s	-0030	110	109	121	105	101	91	99	97	93	135
Mean s	-0020	92	112	74	90	85	84	85	91	96	85
Mean s	-0010	91	96	88	97	105	102	107	118	120	121
Mean s	0000	110	105	97	88	98	103	93	91	86	94
Mean s	0010	96	86	85	84	99	91	123	104	111	118
Mean s	0020	105	92	84	78	89	85	103	103	86	103
Mean s	0030	92	92	102	116	142	125	94	76	88	95
Mean s	0040	91	999								
